# Lifestyle habits and health behaviors in first-year medical students: a longitudinal analysis across the academic year

**DOI:** 10.3389/fspor.2026.1781555

**Published:** 2026-04-08

**Authors:** Giovanna Zimatore, Ludovica Cardinali, Carlo Baldari, Manuela Minozzi, Emiliane Rubat du Mérac, Piercesare Grimaldi, Edoardo Mocini, Silvia Migliaccio, Olivia Di Vincenzo, Laura Guidetti, Mario Cesare Nurchis, Dafne Ferrari, Maria Chiara Gallotta

**Affiliations:** 1 Department of Theoretical and Applied Sciences, DiSTA, eCampus University, Novedrate, Italy; 2 Department of Life Sciences, Health, and Health Professions, Link Campus University, Rome, Italy; 3 Department of Psychology of Development and Socialization Processes, Sapienza University of Rome, Rome, Italy; 4 Department of Experimental Medicine, Sapienza University of Rome, Rome, Italy; 5 Department of Humanities, Movement and Education Sciences, University Niccolò Cusano, Rome, Italy; 6 Department of Physiology and Pharmacology “Vittorio Erspamer”, Sapienza University of Rome, Rome, Italy

**Keywords:** chronic diseases, mediterranean diet, modifiable behaviors, physical activity, psychological well-being, risk factors, university lifestyle

## Abstract

**Background:**

Despite being educated in a health-oriented environment, medical students are vulnerable to developing unhealthy habits that may compromise their well-being and future professional effectiveness. This study aimed to assess changes in lifestyle behaviors and psychological well-being among first-year medical students over one academic year.

**Methods:**

A longitudinal observational study was conducted on a cohort of 189 first-year medical students at Link Campus University (Rome, Italy). Assessments were performed at the beginning and end of the academic year (November 2024–June 2025). A total of 87 students completed all surveys. Measures included self-reported anthropometric data, the Mediterranean Diet Adherence Screener (MEDAS), International Physical Activity Questionnaire (IPAQ), Volition in Exercise Questionnaire (VEQ-I), Perceived Stress Scale (PSS-10), WHO5 Well-Being Index, and the Psychological General Well-Being Index (PGWBIS). Statistical analyses included paired t-tests or Wilcoxon signed-rank tests, and correlation analyses using Spearman's coefficients.

**Results:**

Of the total of sample, 102 students were female (65%) and 55 were male (35%), with median age was 19.8 years old. At follow-up, a statistically significant increase in BMI was observed (*p* = 0.009). No significant changes were found in physical activity levels or Mediterranean diet adherence. However, volitional facilitation for physical activity significantly declined (*p* = 0.020), indicating reduced motivation. Psychological well-being worsened across the academic year, with increased perceived stress (*p* < 0.001), and decreased WHO5 (*p* = 0.046) and PGWBIS scores (*p* = 0.055). Significant correlations were found between psychological well-being and both physical activity and diet adherence.

**Conclusions:**

Over the course of one academic year, medical students reported moderate levels of perceived stress and average emotional and psychological well-being. Despite increasing psychological strain, students maintained a stable core of eating behaviors, suggesting a remarkably resilient and structured dietary pattern. Nevertheless, they exhibited a predictable decline in psychological well-being during examination periods, accompanied by a reduction in motivation for physical activity and modest weight gain. Overall, these findings underscore the importance of targeted interventions within medical education to promote and sustain healthy lifestyle behaviors during the early stages of academic training.

## 1 Introduction

The university period represents a critical transition to adulthood, characterized by behavioral, psychological, and lifestyle changes (1). Particularly, the first academic year marks a pivotal stage of adjustment involving adaptation to a new environment, the development of new social relationships, increased autonomy, and exposure to several academic and psychosocial stressors (2). These include demanding academic workload, time management challenges, academic competition, financial concerns, and family expectations. Such stressors can reduce motivation, impair academic performance, and increase the risk of dropout. Moreover, they are frequently associated with unhealthy lifestyle behaviors, such as alcohol and tobacco use, low physical activity levels, and poor dietary habits (1, 3), that can negatively affect students' physical and psychological well-being (4, 5).

Medical students, despite being immersed in health-oriented environments, are not exempt from these challenges. The academic demands and emotional pressures of medical school may compromise their ability to maintain healthy habits, often leading to maladaptive coping behaviors that negatively affect their physical and psychological well-being (6). Previous studies have shown that medical students frequently experience substantial lifestyle changes and increases in stress, especially during the first year (6–8). These changes are primarily attributed to limited time, poor stress and sleep management, and emerging symptoms of anxiety, depression, or fatigue (6).

However, as future physicians, medical students are expected to have positive attitudes and practice healthy behaviors along with a deeper understanding of the long-term health consequences associated with smoking, excessive alcohol consumption, poor diet and sedentary lifestyles (6). Although medical education has historically been disease-oriented, focusing primarily on diagnosis and treatment, recent developments have increasingly emphasized prevention and well-being. Accordingly, medical training should also foster awareness and self-care habits consistent with health promotion principles.

Notably, our previous findings (9) suggest that greater physical activity engagement and enhanced well-being outcomes were predominantly reported by males and by students outside the medical cohort (non-medical students). These differences suggest that contextual and psychosocial factors play a critical role in shaping students' health-related behaviors and coping strategies.

The personal health behaviors of medical students have implications beyond their own well-being. Evidence indicates that physicians who practice healthy behaviors are more likely to counsel patients effectively and promote positive lifestyle changes (6, 10, 11). Supporting this, data from the Italian PASSI surveillance system (2016–2019) show that adherence to healthy behaviors in the adult population is strongly associated with receiving advice from healthcare professionals (12).

For these reasons, medical schools should play an active role in promoting student health, as defined by the World Health Organization as a state of complete physical, mental, and social well-being, not merely the absence of disease. Understanding the dynamics of lifestyle behaviors and psychological well-being among medical students is essential to designing effective interventions that prevent unhealthy habits and enhance resilience.

The present study aimed to evaluate changes in lifestyle behaviors, including physical activity, adherence to the Mediterranean diet, and psychological well-being, among first-year medical students over the course of one academic year.

## 2 Materials and methods

### 2.1 Design and participants

A longitudinal observational study was conducted at Link Campus University (Rome, Italy) between November 2024 and June 2025. The research protocol was approved by the University Ethics Committee (ID: 542), and all participants provided written informed consent in accordance with the Declaration of Helsinki. The study population included all first-year medical students enrolled in the 2024/2025 academic year. Specifically, during the university courses the authors (G.Z., L.C., M.M., L.G., P.G.) explained the project and invited the student to participate. Participation was voluntary, and data were collected anonymously. Eligibility criteria were: (i) age ≥ 18 years; (ii) attendance during the first semester (November 2024); and (iii) continuous enrollment until the end of the second semester (June 2025). Students who withdrew before follow-up or failed to complete both assessments were excluded. No formal sample size calculation was performed because the study included the entire cohort of first-year medical students enrolled during the academic year.

### 2.2 Outcome measures

Data were collected at two time points: at the beginning (T1, November 2024) and at the end (T2, June 2025) of the academic year, to evaluate longitudinal changes in anthropometric, behavioral, and psychological measures. Students completed a self-administered online questionnaire distributed via Google Forms. The survey link was provided directly during classroom sessions. To encourage participation and minimize dropout rates, the questionnaire was designed to take no longer than 10–15 min to complete. The survey assessed multiple aspects of students' lifestyle behaviors and psychological well-being through a battery of six validated questionnaires. These tools evaluated dietary habits, physical activity levels, volitional motivation for exercise, perceived stress, and psychological well-being.

#### 2.2.1 Anthropometric and socio-demographic measures

Participants self-reported age, sex, weight, and height. Body Mass Index (BMI) was calculated as body weight in kilograms divided by height in meters squared (kg/m²). Cases with missing height or weight values were excluded from BMI-related analyses.

#### 2.2.2 Mediterranean diet adherence (MEDAS)

Adherence to mediterranean diet (MD) was evaluated using the 14-item Mediterranean Diet Adherence Screener (MEDAS) (13).

The questionnaire includes 12 items the frequency of food consumption and 2 items on specific dietary habits related to the MD. Each item scores 1 point if the response meets MD recommendation; otherwise, it received 0 points, resulting in a total score ranging from 0 to 14. Based on the MEDAS score, participants were categorized into three levels of MD adherence: Low (score ≤ 5) Moderate (score between 6 and 9) High (score ≥ 10).

#### 2.2.3 Physical activity levels (IPAQ-SF)

Physical activity levels were assessed using the International Physical Activity Questionnaire—Short Form (IPAQ-SF) (14). This seven-item tool records the frequency (days per week) and duration (minutes per day) of vigorous, moderate, and walking activities over the previous seven days, as well as daily sitting time.

Activity levels were expressed in Metabolic Equivalent of Task (MET)-minutes/week, according to the official IPAQ scoring protocol, using standard conversion factors for each activity: 3.3 METs for walking, 4.0 METs for moderate activity, and 8.0 METs for vigorous activity. Total MET minutes/week were calculated by adding (days × minutes per day × MET) for each intensity level. Based on the total MET-minutes/week, participants were classified into three categories according to IPAQ criteria: (i) Low: Not meeting the criteria for moderate or high activity levels; (ii) Moderate: ≥600 MET-min/week, accumulated over ≥5 days of moderate activity or ≥3 days of vigorous activity or any combination; (iii) High: ≥1,500 MET-min/week of vigorous activity on ≥3 days, or ≥3,000 MET-min/week of any combination of activities on ≥7 days. Sitting time, reported as average daily minutes, was analyzed separately and not included in total physical activity calculations.

#### 2.2.4 Volition in exercise (VEQ-I)

The assessment of volitional factors influencing physical activity behavior was conducted using the Italian version of the Volition in Exercise Questionnaire (VEQ-I) (15). The instrument includes 18 items rated on a 4-point Likert scale (0 = “does not match at all” to 3 = “exactly matches”). It measures six dimensions: four volitional facilitation factors (VI) (Reasons, Postponing Training, Unrelated Thoughts, and Approval from Others), which reflect barriers to pursuing exercise goals, The VEQ-I includes two volitional facilitation factors (VEQI-VF) Self-Confidence and Coping with Failure, which promote goal achievement and sustained participation in physical activity. Conversely, four volitional inhibition factors (VEQI-VI) Reasons, Postponing Training, Unrelated Thoughts, and Approval from Others represent barriers that hinder goal pursuit. Each subscale ranges from 0 to 3. A global volitional score can be obtained by calculating the difference between the mean VF and VI scores (VF—VI), yielding values from −3 to +3. Positive scores indicate that volitional facilitation predominates over inhibition, reflecting stronger self-regulatory and motivational resources, whereas negative scores suggest that inhibitory tendencies outweigh facilitative processes, implying greater difficulty in maintaining exercise engagement.

#### 2.2.5 Perceived stress scale (PSS-10)

The Italian version of the Perceived Stress Scale (PSS-10) was used to assess the extent to which individuals perceived situations in their lives as stressful during the past month (16). This self-report instrument consists of 10 items designed to measure how unpredictable, uncontrollable, and overloaded respondents perceive their lives. Each item is rated on a 5-point Likert scale ranging from 0 (“never”) to 4 (“very often”), indicating how frequently the respondent has experienced certain feelings or thoughts in the past month. The scale includes six negatively worded items (items 1, 2, 3, 6, 9, and 10), which reflect perceived distress, and four positively worded items (items 4, 5, 7, and 8), which reflect perceived coping. Positive items are reverse scored during analysis. The total score ranges from 0 to 40, with higher scores indicating greater levels of perceived stress.

#### 2.2.6 World health organization—five well-being index (WHO5)

Subjective psychological well-being was assessed with the World Health Organization Five Well-Being Index (WHO5) (17). It comprises five items through which individuals report how they have felt over the past two weeks, using a six-point Likert scale ranging from 0 (“at no time”) to 5 (“all of the time”). The items address key aspects of emotional well-being, including feeling calm and relaxed, energy levels, interest in daily activities, happiness, and waking up feeling refreshed. Total scores range from 0 to 25, with higher scores indicating better psychological well-being and lower scores suggesting reduced mental health.

#### 2.2.7 Psychological general well-being index (PGWBIS)

The short version of the Psychological General Well-Being Index (PGWBIS) (18–20) was used to assess individuals' self-perceived psychological well-being and levels of distress. The scale includes six items, each representing one of the following domains: anxiety, depressed mood, positive well-being, self-control, general health, and vitality. It offers a measure of both the positive and negative facets of well-being reported in the past month. Each item is rated on a six-point Likert scale ranging from 0 (“none of the time”) to 5 (“all of the time”), yielding a total score between 0 and 30. Higher total scores indicate greater psychological well-being.

### 2.3 Statistical analysis

Descriptive statistics were calculated for all variables, including means, standard deviations, medians, and interquartile ranges as appropriate. The normality of data distribution was assessed using the Shapiro–Wilk test and visual inspection of histograms and Q-Q plots.

An attrition analysis was conducted using independent-samples t-tests on baseline demographic, anthropometric, behavioral and psychological variables, to compare participants who completed the study and those who dropped out. Depending on the distribution, correlation analyses were conducted using Pearson's or Spearman's rank correlation coefficients to examine relationships between change scores of all outcome variables.

The correlation matrix was generated to explore potential associations between different domains of change associated with attendance of first-year medical courses.

For pre-post intervention comparisons, parametric or non-parametric tests were selected based on the normality of the data distribution as well. Paired *t*-tests were used for normally distributed variables, while the Wilcoxon signed-rank test was applied for non-normally distributed variables. Effect sizes were calculated where appropriate through the Cohen's d or the rank-based correlation r. Effect size for parametric tests (i.e., Cohen's d) is interpreted as small (0.2), medium (0.5), large (0.8). Effect size for nonparametric tests (i.e., rank based correlation r) is interpreted as small (0.1), medium (0.3), large (0.5). The strength of correlations was interpreted according to previously published guidelines (21).

All statistical tests were two-tailed, and *p*-values < 0.05 were considered statistically significant. Missing data were handled using pairwise deletion for correlation analyses, and complete case analysis was performed for pre-post comparisons. Statistical analyses were performed using STATA version 17.0 (StataCorp, College Station, TX) and R version 4.5.1 (R Core Team, R Foundation for Statistical Computing, Austria).

## 3 Results

### 3.1 Sample characteristics

A total of 189 students were initially invited to participate in the study. Of these, 157 (83%) completed the baseline assessment; Eighty-seven students completed both time points, corresponding to a retention rate of 55%, and constituted the sample used for the T1–T2 comparison. The remaining 70 students (45%) did not complete the follow-up assessment and were included in the attrition analysis. Among the completers (*n* = 87), 63% were female (*n* = 55) and 37% were male (*n* = 32), with a mean age of 19.0 ± 2.0 years. Among the dropouts (*n* = 70), 67% were female (*n* = 47) and 33% were male (*n* = 23), with a mean age of 20.5 ± 3.2 years.

Descriptive statistics for anthropometric, behavioral, and psychological measures at baseline (T1) and follow-up (T2) are summarized in Table 1.

**Table 1:** Baseline (T1) and follow-up (T2) characteristics of first-year medical students (*n* = 87).

Type	Variable	T1 (mean ± SD)/median (IQR)	T2 (mean ± SD)/median (IQR)	t/Z	*p*	d/r	95% CI
Anthropometric	Height (cm)	169.9 ± 0.9	169.9 ± 0.9	t = −0.45	0.657	−0.05	−0.26–0.16
	**Weight (kg)**	**65.1** ± **12.8**	**66.1** ± **12.6**	**t** **=** **2.68**	**0**.009**	**0**.**293**	**0.08–0.50**
	**BMI (kg/m²)**	**22.01 (4.32)**	**22.41 (4.55)**	**Z** **=** **−2.60**	**0**.009**	**0**.**279**	**0.07–0.46**
Behavioral	MEDAS	6.67 ± 1.92	6.83 ± 1.90	t = −0.79	0.434	0.161	0.07–0.46
	IPAQ (MET·min/week)	2,613 (3,804)	2,970 (4,452)	Z = −0.86	0.389	0.092	−0.12–0.30
	**VEQI-VF**	**1.91** ± **0.80**	**1.63** ± **0.77**	**t** **=** **2.38**	**0**.020*	**−0**.**255**	**−0.47–−0.04**
	VEQI-VI	1.20 ± 0.60	1.10 ± 0.60	t = 1.39	0.168	−0.149	−0.36–0.06
	VEQ-I (VF–VI)	0.61 ± 0.90	0.58 ± 0.80	t = 0.32	0.752	−0.034	−0.24–0.18
Psychological Well-Being	**PSS−10**	**18.7** ± **5.8**	**21.5** ± **4.5**	**t** **=** **–3.82**	**<0**.**001**	**0**.**409**	**0.18–0.64**
	**WHO5**	**13.2** ± **4.5**	**12.3** ± **4.5**	**t** **=** **2.02**	**0**.046*	**−0**.**217**	**−0.43–−0.02**
	PGWBIS	17.4 ± 4.8	16.5 ± 4.8	t = 1.95	0.055	−0.209	−0.42–0.01

BMI, body mass index; MEDAS, mediterranean diet adherence screener; IPAQ, international physical activity questionnaire; VEQ-I, volition in exercise questionnaire; VF, volitional facilitation; VI, volitional inhibition; PSS-10, perceived stress scale; WHO5, five well-being index; PGWBIS, psychological general well-being index.

*p <0.05, and **p <0.01 are in bold.

### 3.2 Attrition analysis

An attrition analysis was conducted to compare completers (*n* = 87) and dropouts (*n* = 70) on baseline (T1) demographic, anthropometric, behavioral and psychological variables using independent-samples *t*-tests. The two groups were largely comparable at baseline, however, as shown in Table 2, dropouts reported higher MEDAS scores (*p* = .002) and greater WHO5 well-being scores (*p* = .026).

**Table 2:** Independent-samples t-tests comparing baseline characteristics of completers and dropouts.

Type	Variable	Completers (*n* = 87) (mean ± SD)	Dropouts (*n* = 70) (mean ± SD)	t	*p*	d	95% CI
Anthropometric	**Age (years)**	**19.31** ± **2.00**	**20.44** ± **3.25**	**−2**.**556**	**0**.012*	**−0**.**431**	**[−2.0; −0.26]**
	Weight (kg)	65.09 ± 12.79	70.11 ± 20.08	−1.769	0.065	−0.298	[−10.59; 0.54]
	Height (cm)	169.9 ± 8.9	179.2 ± 9.3	−0.267	0.789	−0.043	[−3.27; 2.49]
	BMI (kg/m²)	22.5 ± 3.4	24.0 ± 6.3	−1.818	0.056	−0.31	[−3.16; 0.12]
Behavioral	**MEDAS**	**6.7** ± **1.9**	**7.7** ± **2.1**	**−3**.**073**	**0**.002**	**−0**.**499**	**[−1.65; −0.36]**
	IPAQ (METs)	3,351 ± 3,170	4,323 ± 4,240	−1.597	0.102	−0.264	[−2,170; 222]
	VEQI-VF	1.73 ± 0.75	1.58 ± 0.76	1.237	0.218	0.199	[−0.09; 0.39]
	VEQI-VI	1.01 ± 0.52	1–03 ± 0.57	−0.181	0.8567	−0.029	[−0.19; 0.16]
	VEQ-I (VF–VI)	0.61 ± 0.94	0.86 ± 0.81	−1.772	0.083	−0.28	[−0.52; 0.03]
Psychological Well-Being	PSS-10	18.7 ± 5.8	17.0 ± 6.1	1.782	0.075	0.288	[−0.17; 3.58]
	**WHO5**	**13.2** ± **4.5**	**14.9** ± **5.2**	**−2**.**21**	**0**.026*	**−0**.**36**	**[−3.3; −0.2]**
	PGWBIS	17.4 ± 4.8	18.6 ± 4.3	−1.683	0.099	−0.267	[−2.64; 0.2]

BMI, body mass index; MEDAS, mediterranean diet adherence screener; IPAQ, international physical activity questionnaire; VEQ-I, volition in exercise questionnaire; VF, volitional facilitation; VI, volitional inhibition; PSS-10, perceived stress scale; WHO5, five well-being index; PGWBIS, psychological general well-being index.

*p <0.05, and **p <0.01 are in bold.

Participants in the dropouts group were significantly older (*t* = −2.56, *p* = 0.012, Cohen's d = −0.43) and reported significantly higher MEDAS quality scores (*t* = −3.07, *p* = 0.0025, *d* = −0.50), as well as significantly higher psychological well-being (WHO5) (*t* = −2.21, *p* = 0.029, *d* = −0.36); no other significant differences emerged for the remaining variables. The effect sizes associated with the significant comparisons ranged from small-to-moderate magnitude. Specifically, the difference in age (*d* = −0.43) and psychological well-being (WHO5) (*d* = −0.36) reflects a small-to-moderate effect, indicating meaningful but not large group differences. The effect size for diet quality (*d* = −0.50) reached a moderate magnitude, suggesting a more substantial practical difference between the two groups. All 95% confidence intervals associated with the significant effects did not include zero, confirming the statistical robustness and reliability of the observed group differences. Moreover, the relatively narrow width of the intervals indicates a good precision of the estimates, supporting the stability of the effect size and mean difference values.

Since, among the variables that showed significant pre–post differences, only the WHO5 score also differed between the Completers group and the Dropout group the longitudinal analyses can be considered largely unaffected by dropout-related bias, as confirmed by the attrition test, However, findings related to WHO5 should be interpreted with caution. Participants who remained in the study had lower baseline WHO5 scores and exhibited a further decline at follow-up, suggesting that the observed changes in psychological well-being may partially reflect selective retention rather than true longitudinal change.

### 3.3 Longitudinal changes across the academic year

#### 3.3.1 Anthropometric

A significant increase in BMI was observed across the academic year (*Z* = −2.598, *p* = 0.009, *r* = 0.279). Overall, 59.8% of students showed BMI increases, suggesting modest weight gain during the transition to university life.

#### 3.3.2 Behavioral outcomes

**Figure 1:**
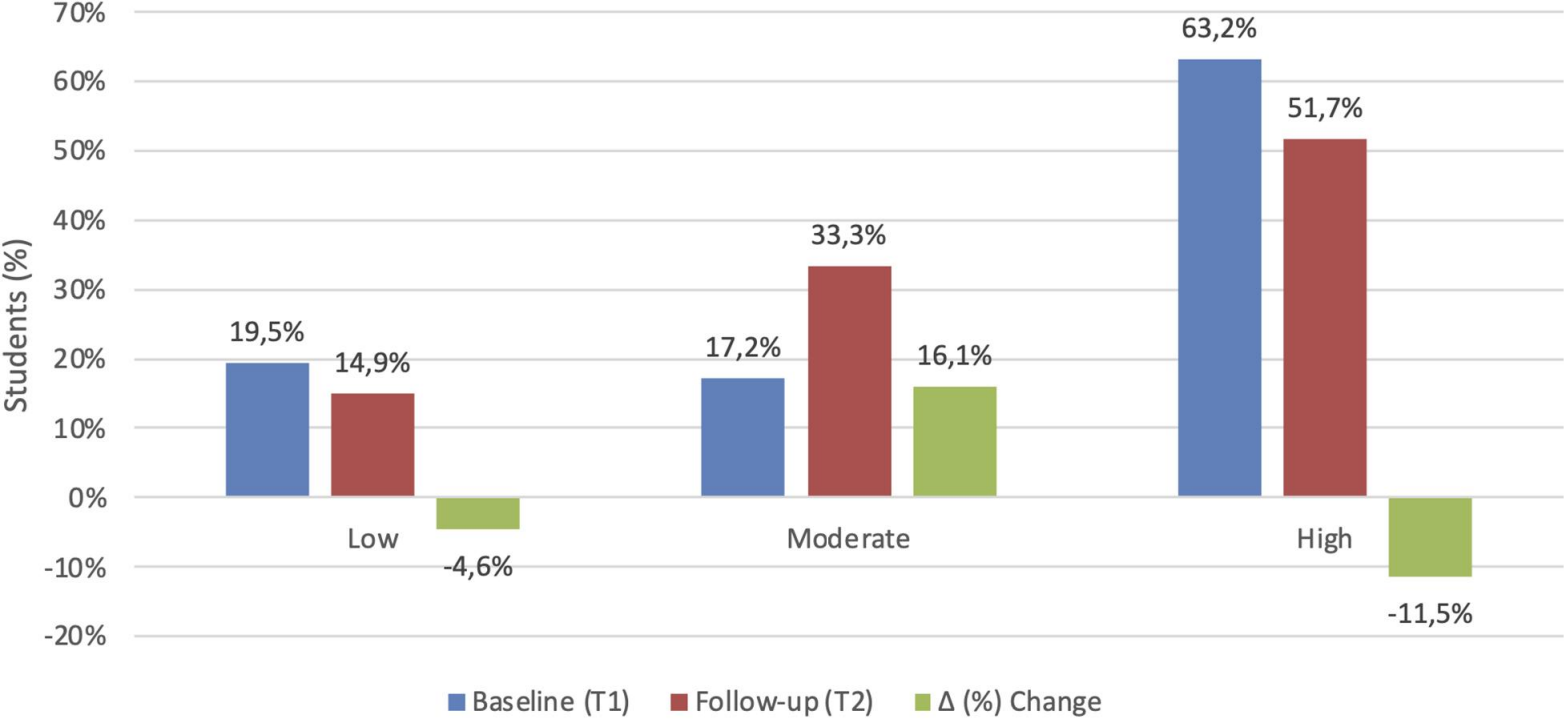
Distribution of IPAQ physical activity categories (%) at baseline (T1) and follow-up (T2) (*n* = 87).

The distribution of participants across IPAQ physical activity categories is reported in Figure 1 for descriptive purposes. Although some shifts in category distribution were observed, none of the changes were statistically significant. Physical activity levels (IPAQ MET-min/week) remained stable across the academic year (*Z* = −0.861, *p* = 0.389, *r* = 0.092), indicating no significant change in overall activity.

When examining physical activity categories (Low, Moderate, High) using Bowker's test of symmetry, the change in distribution was not statistically significant (*χ*^2^ = 6.22, df = 3, *p* = 0.101).

Volitional facilitation (VEQI-VF), reflecting intrinsic motivation and self-confidence in maintaining physical activity, significantly declined from baseline to follow-up (*t* = 2.38, *p* = 0.020, *d* = −0.255). In contrast, volitional inhibition (VEQI-VI) (*t* = 1.39, *p* = 0.168, *d* = −0.149) and the composite VEQ index (*t* = 0.32, *p* = 0.752, *d* = −0.034) remained stable. These findings indicate that while perceived barriers to exercise did not increase, students experienced a decline in intrinsic motivation and self-confidence toward exercise to remain active toward the end of their academic year.

Analysis of dietary habits revealed stable adherence to the Mediterranean diet (MEDAS score) across the academic year (*t* = −0.789, *p* = 0.434, *d* = 0.161). Mean scores remained within the moderate adherence range (T1 = 6.67 ± 1.92; T2 = 6.83 ± 1.90), suggesting that dietary patterns were relatively consistent despite increased academic demands.

#### 3.3.3 Psychological well-being

Students reported significantly higher perceived stress (PSS-10) (*t* = –3.82, *p* < 0.001, *d* = 0.409) and lower subjective well-being on both WHO5 (*t* = 2.02, *p* = 0.046, *d* = −0.217) and PGWBIS (*t* = 1.95, *p* = 0.055, *d* = −0.209). These results indicate a pattern of deterioration in psychological health toward the end of the first academic year.

### 3.4 Gender-based analysis

Gender-based comparisons indicated that male students exhibited a greater increase in perceived stress (PSS10) than females (*t* = –1.92, *p* = 0.059), approaching statistical significance. No gender differences were found in changes in WHO5 (*t* = –0.41, *p* = 0.685), PGWBS (*t* = –1.44, *p* = 0.154), VEQI_VF (*t* = 0.13, *p* = 0.897), VEQI_VI (*t* = 0.32, *p* = 0.750), IPAQ (*t* = 0.93, *p* = 0.357), or MEDAS (*t* = 0.02, *p* = 0.986). These results suggest that, while both sexes experienced similar behavioral and psychological trends, male students may have been more vulnerable to increased stress during the first academic year.

### 3.5 Correlations between behavioral and psychological variables

To explore the relationships among Behavioral and psychological well-being, pairwise Spearman correlations were computed separately for baseline and follow-up assessments.

Overall, consistent associations emerged, showing that higher stress levels were inversely related to well-being, while greater adherence to healthy habits (diet and exercise) correlated positively with psychological outcomes.

#### 3.5.1 Baseline correlations

**Figure 2:**
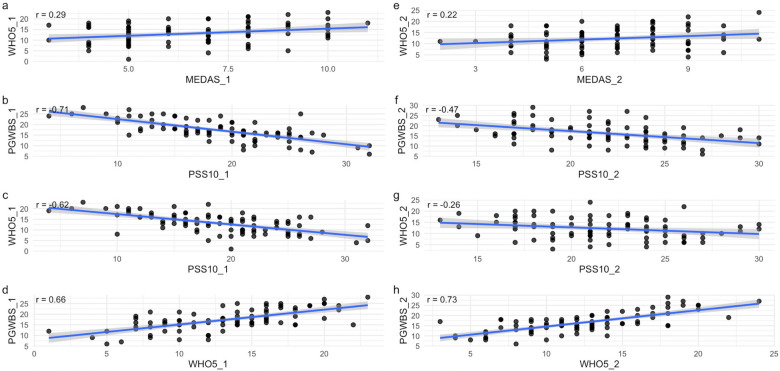
Scatter plots illustrate significant spearman correlations (*p* < .05) between behavioral and psychological variables on the left at baseline (T1) (*n* = 87) **(**panel **a–d)** and on the right at follow up (T2) **(e–h)**. All associations are significant at the 5% level (α = 0.05). MEDAS, mediterranean diet adherence screener; PSS-10, perceived stress scale; WHO5, five well-being index; PGWBIS, psychological general well-being index. Variables labeled with the suffix “_1” correspond to baseline (T1), whereas those labeled “_2” refer to follow-up (T2).

At baseline, several significant associations were observed among behavioural and psychological variables (Figure 2, left panels).

At the 10% significance level (α = 0.10), Perceived stress (PSS-10) showed a moderate negative correlation with WHO5 (*r* = –0.622), and a strong negative correlation with PGWBIS (*r* = –0.713), indicating that higher perceived stress was associated with lower levels of psychological well-being. PSS-10 also showed a weak negative correlation with physical activity (IPAQ; r = –0.195), indicating that students who perceived higher stress could be less physically active. Psychological well-being (WHO5) was weakly positively associated with both MEDAS (*r* = 0.282) and IPAQ (*r* = 0.186, weak), suggesting that greater well-being could correspond to healthier dietary patterns and higher physical activity. Similarly, general well-being (PGWBIS) was weakly positively correlated with both MEDAS (*r* = 0.166), IPAQ (*r* = 0.183), and moderatly positively correlated WHO5 (*r* = 0.658), ndicating coherence between different well-being measures and lifestyle behaviours.

Volitional facilitation (VEQI-VF) was weakly negatively correlated with age (*r* = –0.181).

At the more stringent 5% significance level (α = 0.05), the strongest associations remained statistically significant. PSS-10 maintained moderate to strong negative correlations with WHO5 (*r* = −0.622) and PGWBIS (*r* = −0.713), confirming the inverse relationship between perceived stress and psychological well-being. WHO5 also remained weakly positively correlated with MEDAS (*r* = 0.282), while PGWBIS and WHO5 continued to show a moderate positive correlation (*r* = 0.658), reinforcing the internal coherence among well-being indicators and highlighting the potential contribution of healthy dietary patterns to psychological health.

#### 3.5.2 Follow-up correlations

**Figure 3:**
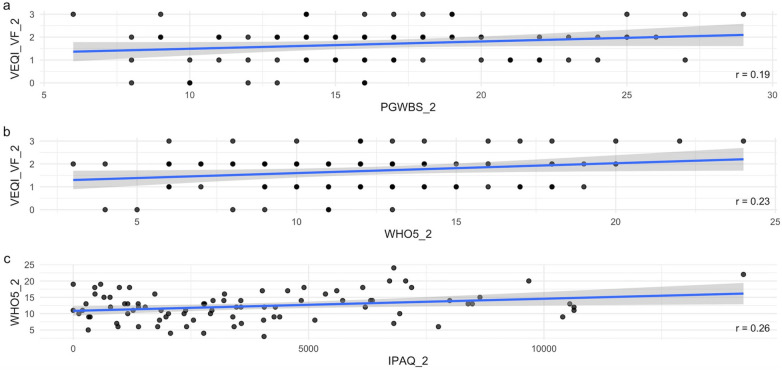
Scatter plots illustrating significant spearman correlations (*p* < .05) between behavioral and psychological variables at follow-up (T2) (*n* = 87) (panel **a**–**c**); VEQ-I, volition in exercise questionnaire; VF, volitional facilitation; VI, volitional inhibition; WHO5, five well-being index; PGWBIS, psychological general wellbeing index; IPAQ, international physical activity questionnaire. Variables labeled with the suffix “_1” correspond to baseline (T1), whereas those labeled “_2” refer to follow-up (T2).

The follow-up correlation analysis also identified several statistically significant associations between motivation, well-being, and health behaviours (Figure 2, right panels; Figure 3).

At the 10% significance level (α = 0.10), WHO5 showed weak positive correlation with MEDAS (*r* = 0.211), IPAQ (*r* = 0.238), and volitional facilitation (VEQI-VF, *r* = 0.306), and weak negative correlation with PSS-10 (*r* = –0.308).

PGWBIS showed a strong positive correlation with WHO5 (*r* = 0.725) and a weak positive correction with VEQI-VF (*r* = 0.265), and weak negative correlation with PSS-10 (*r* = –0.397), highlighting the inverse relationship between stress and well-being. In addition, VEQI-VF showed a weak positive correlation with IPAQ (*r* = 0.192) and WHO5 (*r* = 0.306), supporting the notion that higher volitional facilitation could be linked to sustained engagement in physical activity and better psychological functioning.

At the more stringent 5% significance level (α = 0.05), several associations remained significant. PSS-10 maintained weak negative correlations with WHO5 (*r* = –0.308) and PGWBIS (*r* = –0.397), indicating that the higher perceived stress was associated with lower levels of well-being.

PGWBIS and WHO5 remained strongly positively correlated (*r* = 0.725). WHO5 also preserved weak positive correlations with IPAQ (*r* = 0.238) and VEQI-VF (*r* = 0.306). Finally, MEDAS showed a weak positive correlation with WHO5 (*r* = 0.211), while PGWBS was remained positively correlated with VEQI-VF (*r* = 0.265).

In summary, the four significant correlations found both at baseline and at follow up involved psychological variables, whereas the three associations that emerged exclusively at follow-up reflected the role of experience and volitional engagement in physical activity.

## 4 Discussion

This longitudinal observational study examined changes in lifestyle behaviors and psychological well-being among medical students across their first academic year. The present investigation builds upon our previous cross-sectional study conducted among medical and non-medical university students, which explored the relationships between lifestyle, volitional factors, and psychological health (9). Understanding these domains in this population provides a critical evidence base for designing and implementing interventions that foster the adoption and maintenance of healthy habits during university years, while also strengthening future physicians' willingness and capacity to promote healthy lifestyles to their patients. In line with our previous findings (9), which emphasized the pivotal role of volitional processes and psychological well-being in shaping university students' health behaviors, the present results confirm the relevance of these mechanisms in sustaining or hindering healthy routines under academic pressure. Our earlier work identified gender- and faculty-related differences, with males and non-medical students showing higher physical activity and better well-being; the current data extend this evidence to medical students, who appear particularly vulnerable to psychological strain and reduced volitional facilitation for exercise. The main findings of our study revealed a modest but meaningful deterioration in psychological well-being and an increase in BMI across the academic year among medical students, with a stable level of physical activity and diet but less volitional facilitation for exercise.

### 4.1 Anthropometrics

The observed BMI increase over the academic year indicates a modest but measurable trend toward weight gain among first-year medical students over the course of the academic year. This result aligns with previous longitudinal studies reporting weight gain and increases in BMI during the transition to university life, particularly in the first year, due to the combined effects of environmental, behavioral, and psychological stressors (22, 23). In particular, Vadeboncoeur et al. (23) conducted a large meta-analysis aimed at examining the so-called “Freshman 15” phenomenon, a widely held belief that students gain an average of 15 pounds (6.8 kg) during their first year of university. Their findings demonstrate that while this notion is largely a myth at the population level, weight gain during the first year is nonetheless common and clinically relevant. Specifically, the meta-analysis of 32 studies involving 5,549 students found an average weight gain of 1.36 kg over approximately five months of the first year, with 60.9% of students gaining weight. Among those who did gain weight, the average increase was 3.38 kg, indicating the phenomenon is concentrated in a vulnerable subgroup. Approximately 9.3% of students gained at least 6.8 kg, representing a clinically significant risk. Vadeboncoeur et al. (23) further identified that most weight gain occurs during the first semester, highlighting this initial period as particularly critical. This timing suggests that the sudden transition to university life characterized by new academic pressures, lifestyle adjustments, decreased physical activity, changed eating patterns, and heightened stress, may be pivotal in determining students' weight trajectories.

Despite the observed weight gain over the academic year, the results of our study showed no significant changes in overall physical activity levels or adherence to the Mediterranean diet as described in the following paragraphs.

### 4.2 Physical activity

Recent studies focusing on university populations, particularly medical students, have highlighted the important role of physical activity in supporting both physical and psychological health during higher education. Evidence suggests that the transition to university is often associated with changes in physical activity patterns, with many students experiencing reductions in activity levels as academic demands increase (6, 24). Among medical students, the intensive academic workload and time constraints may further contribute to difficulties in maintaining regular physical activity throughout the academic year (7). Nevertheless, physical activity has consistently been identified as a protective factor for mental health in university populations, being associated with lower levels of stress, anxiety, and depressive symptoms (4, 25). Within this framework, examining physical activity patterns during the first year of medical school is particularly relevant, as this period represents a critical transition in which lifestyle behaviors may change in response to academic demands.

However, longitudinal evidence examining changes in physical activity during the first year of medical school remains limited. Understanding how physical activity patterns evolve during this critical transition period may help clarify how academic demands interact with students' health behaviors. Across age groups, physical activity consistently appears as a protective factor, associated with better psychological and cognitive outcomes, while unhealthy lifestyle patterns are linked to poorer functioning. In primary school children, appropriately designed physical education lessons were shown to enhance attention and concentration and to modulate physiological stress responses, with lesson structure and timing emerging as important moderators of these effects (26). Similarly, in the university context, maintaining adequate levels of physical activity may contribute to preserving psychological well-being and motivational resources during periods of increased academic pressure.

Within this framework, the associations observed in our study between psychological well-being, physical activity, and motivational factors gain further relevance. Our findings suggest continuity in the role of physical activity as a protective lifestyle behavior across the educational lifespan, reinforcing the notion that interventions aimed at promoting active behaviors may have beneficial effects not only on physical health, but also on psychological resilience and academic-related outcomes. Within this lifespan-oriented framework, recent evidence from tele-exercise interventions further supports the interpretation of physical activity as an adaptive and protective resource (27) showed that participation in structured tele-exercise programs was associated with significant improvements in psychological well-being even in the presence of increased perceived stress, suggesting that physical activity may enhance individuals' capacity to cope with stress rather than merely reduce it. These findings should be interpreted within the broader literature suggesting that physical activity may represent a potential resource for supporting psychological well-being in demanding academic contexts.

Regarding physical activity levels (measured in MET-min/week using the IPAQ), our study found no statistically significant changes from the beginning to the end of the academic year. These results differ from those reported in a previous study, which observed a decrease of 147.9 total MET-min/week after one year of follow-up from university admission (24). In our sample, the distribution of physical activity categories also did not show statistically significant changes across the academic year.

A gradual decline in structured or vigorous physical activity probably due to increasing time constraints and academic workload is consistent with the framework proposed by Quinzi et al. (28), who emphasized that physical activity engagement in university students is strongly influenced by volitional processes rather than by environmental context alone. In line with this interpretation, a notable finding of the present study was the significant decline in volitional facilitation (VEQI-VF) toward the end of their academic year, indicating reduced internal motivation and self-confidence in maintaining regular physical activity. Conversely, volitional inhibition (VEQI-VI), which reflects perceived barriers such as postponing training or cognitive distractions, did not significantly change. As suggested by Quinzi et al. (28), this pattern implies that disengagement from physical activity may be driven more by a weakening of internal self-regulatory resources than by an increase in external or situational obstacles. Overall, these results highlight the importance of reinforcing self-regulatory and motivational skills to support long-term physical activity adherence in this population.

### 4.3 Dietary habits

Adherence to the Mediterranean diet, assessed through the MEDAS score, remained essentially stable across the academic year, with mean values showing no meaningful change between baseline and follow-up (T1 = 6.67; T2 = 6.83). This finding is consistent with longitudinal evidence indicating that dietary habits among university students tend to remain relatively unchanged toward the end of their academic year in the absence of targeted interventions. For instance, Yildiz et al. (29) reported no significant improvement in Mediterranean diet adherence among university students during follow-up, suggesting that eating patterns established at university entry are likely to persist throughout the academic trajectory. In absolute terms, the MEDAS values observed in our sample fall within the lower range of moderate adherence, indicating partial but incomplete alignment with the Mediterranean dietary model. It indicates adequate consumption of certain key components, such as fruits, vegetables and extra-virgin olive oil, but insufficient intake of other essential elements, including legumes, fish and nuts. In a Mediterranean population, and particularly among medical students, such levels should be considered modest and indicative of substantial room for improvement. Our findings are also partially consistent with those reported by Castro-Cuesta et al. (30). In their cross-sectional analysis of Spanish university students, the authors also identified low-to-moderate adherence to the Mediterranean diet, with a mean score of 4.9 ± 1.2 out of 10. Although the average MEDAS score in our sample was slightly higher, both studies converge in highlighting suboptimal dietary patterns among young adults in higher education, even in countries traditionally associated with Mediterranean eating habits. Similar levels of adherence have been reported in other Mediterranean university populations, with mean scores clustering around six points, suggesting that moderate adherence is a common and persistent pattern (31).

This lack of significant change may reflect relatively stable dietary habits among students with moderate baseline adherence. Alternatively, it could indicate limited awareness or insufficient nutritional education to promote meaningful improvements in dietary patterns, despite training in a medical environment.

The correlational analysis provides additional descriptive insight. Higher adherence to the Mediterranean diet showed weak positive associations with psychological well-being at baseline (WHO5: *r* = 0.282; PGWBIS: *r* = 0.166) and remained weakly associated with WHO5 at follow-up (*r* = 0.211). Given the small magnitude of these coefficients, these relationships should be interpreted cautiously, as dietary adherence likely represents only one of several factors contributing to variations in psychological well-being. Overall, these findings are consistent with previous literature suggesting a possible association between healthier dietary patterns and better emotional and psychological functioning.

Moreover, the direction of these associations cannot be determined. It is possible that students with greater personal resources, more effective coping strategies, or more supportive social environments are better able to maintain healthier eating habits while also reporting higher psychological well-being and lower stress levels. In this perspective, dietary habits, physical activity, and psychological well-being may represent related aspects of students' overall lifestyle rather than independent domains linked by a single underlying mechanism.

Notably, despite the increase in perceived stress observed across the academic year (T1 = 18.74; T2 = 21.45), adherence to the Mediterranean diet remained largely unchanged (T1 = 6.67; T2 = 6.83). This finding may suggest that dietary habits in this cohort remained relatively stable during the study period and did not appear to vary substantially in parallel with the observed changes in psychological well-being. However, given the observational design of the study and the limited follow-up period, these findings should be interpreted cautiously, as dietary behaviors are likely influenced by multiple factors beyond short-term fluctuations in perceived stress.

### 4.4 Psychological outcomes

Psychological well-being measures revealed a concerning trend. Perceived stress (PSS-10) significantly increased over the academic year, while both the WHO5 Well-Being Index and PGWBIS scores declined. These findings are consistent with a growing body of evidence indicating that medical students are at increased risk for mental health deterioration during their training, particularly in the early stages (32). The academic and psychosocial pressures inherent to medical education such as high expectations, competitive environments, and reduced leisure time, likely contribute to elevated stress and reduced psychological well-being.

Notably, the inverse correlations between perceived stress and both well-being scores (WHO5 and PGWBIS) support the interdependence of these variables, emphasizing the need for early preventive strategies to promote resilience and mental health among medical students. Moreover, the associations between psychological well-being and both physical activity and diet adherence suggest that lifestyle behaviors may play a protective role and should be encouraged as part of a comprehensive wellness program (33).

The inverse correlations between stress and well-being (WHO5, PGWBIS) at both time points underscore the need for early preventive strategies. Positive associations between well-being, physical activity, and diet adherence align with reciprocal relationships proposed in theoretical models, though causal directionality cannot be inferred. These patterns support multicomponent behavioral and psychological interventions. Moreover, it is important to underline that statistical significance reflects the probability that an observed difference is not due to random variation (34), whereas clinical significance concerns the magnitude and relevance of that difference in real-world or clinical contexts. Therefore, statistically significant findings do not necessarily imply meaningful clinical benefits.

Attrition analysis indicated that participants who remained in the study had lower baseline WHO5 scores and showed a further deterioration at T2. This pattern suggests a potential selective retention of participants with poorer initial well-being, which may have amplified the observed decline in psychological well-being toward the end of their academic year. Consequently, changes in WHO5 should be interpreted with particular caution, as they may partially reflect the effect of attrition rather than a true longitudinal worsening at the population level.

In summary, from a longitudinal perspective, the pattern of correlations indicates that psychological variables remained strongly interrelated across time, whereas the associations involving experience and volitional components of physical activity emerged specifically at follow-up. This shift suggests that, as the academic year progress and academic pressure increased particularly during examination periods motivational and experiential factors related to exercise became more critical in sustaining both physical activity and psychological well-being. In the context of the observed decline in physical activity and well-being at follow-up, these findings support the interpretation that volitional resources may act as a protective mechanism, helping some students to counterbalance the negative impact of sustained academic stress on mental health and lifestyle behaviors.

## 5 Conclusions

Across one academic year, first-year medical students exhibited moderate perceived stress levels and generally preserved emotional and psychological well-being. Eating habits remained largely stable toward the end of the academic year, even under increasing academic demands, suggesting a relatively structured dietary pattern. Since follow-up data were collected close to the examination period, the observed changes may partly reflect contextual academic stress rather than a gradual deterioration occurring throughout the year. Nevertheless, the period toward the end of the academic year was associated with lower psychological well-being, reduced motivation for physical activity, and a modest increase in body weight.

Taken together, these findings highlight that, while certain lifestyle behaviors show resilience, others are highly sensitive to academic stress, underscoring the need for structured, multidimensional intervention strategies within medical education. These should include early stress-management programs, promotion of regular physical activity, nutritional counseling, peer-support initiatives, and the integration of well-being modules into the curriculum, in order to promote sustainable healthy behaviors and effectively protect students' mental and physical well-being during the initial phases of training.

### 5.1 Limitations

This study presents several limitations that should be considered when interpreting the findings.

First, the sample size was relatively small and recruited from a single institution, which may restrict the generalizability of the results to other medical schools or cultural contexts. Second, the study lacked a control group of non-medical students, preventing direct comparisons between medical and non-medical populations and limiting the ability to attribute observed changes specifically to medical education. Third, all measures were based on self-reported questionnaires, which, although validated, are subject to recall bias and social desirability bias. Fourth, due to attrition across the academic year, comparisons between completers and dropouts may have introduced selection bias, as students who withdrew from the study reported higher baseline well-being and dietary adherence.

In addition, the absence of objective assessments of physical activity (e.g., accelerometry) represents a further limitation, as self-reported instruments such as the IPAQ-SF have shown limited agreement with objective measures and tend to substantially overestimate by (14) physical activity levels, potentially affecting the accuracy of lifestyle behavior estimates.

### 5.2 Future perspectives

These results are the basis for new focus and investigations to multi-center cohorts, extending follow up beyond the first academic year. Future research should investigate additional lifestyle factors and evaluate targeted intervention strategies, including structured wellness programs and stress management training. Finally, exploring the role of personality traits and environmental factors may be useful to identify subgroups at higher risk, leading to tailored preventive approaches.

Given that the one-year observational period reflects only short-term changes, future longitudinal multi-institutional studies with longer follow-up and comparison groups are needed to clarify the long-term trajectory of health behaviors and psychological well-being during medical education.

## Data Availability

The raw data supporting the conclusions of this article will be made available by the authors, without undue reservation.

## Appendix A

**Table A1:** Summary of significant correlations at baseline and follow-up.

Variables	Baseline (T1) r	Strength	Follow-up (T2) r	Strength
PSS-10—WHO5	−0.622	Moderate	−0.308	Weak
PSS-10—PGWBIS	−0.713	Strong	−0.397	Weak
WHO5—MEDAS	0.282	Weak	0.211	Weak
WHO5—IPAQ	0.186	Weak	0.238	Weak
PGWBIS—WHO5	0.658	Moderate	0.725	Strong
PGWBIS—MEDAS	0.166	Weak	—	—
PGWBIS—IPAQ	0.183	Weak	—	—
VEQI-VF—WHO5	—	—	0.306	Weak
VEQI-VF—IPAQ	—	—	0.192	Weak
VEQI-VF—PGWBIS	—	—	0.265	Weak

Strength classification based on **Dancey & Reidy** (Questionnaires can be found in 13, 14, 16, 20, 22 and 35).
